# LC–MS/MS-based simultaneous quantification of acylcarnitines, eicosapentaenoic acid, and docosahexaenoic acid: exploring potential biomarkers in human plasma

**DOI:** 10.1007/s00216-025-05943-8

**Published:** 2025-06-07

**Authors:** Baiba Gukalova, Kristaps Krims-Davis, Eduards Sevostjanovs, Aiga Leduskrasta, Ilze Konrade, Maija Dambrova, Edgars Liepinsh

**Affiliations:** 1 Latvian Institute of Organic Synthesis, Aizkraukles Str 21, Riga, LV1006 Latvia; 2 Riga Stradins University, Dzirciema Str 16, Riga, LV1007 Latvia

**Keywords:** Eicosapentaenoic acid, Docosahexaenoic acid, Eicosapentaenoyl-L-carnitine, Docosahexaenoyl-L-carnitine, Acylcarnitines, LC–MS/MS

## Abstract

Acylcarnitines have emerged as valuable markers of the intracellular fatty acid content, mitochondrial functionality, and fatty acid metabolism. However, acylcarnitines derived from polyunsaturated fatty acids (PUFAs) have not been previously examined. To address the need for monitoring lifestyle intervention and omega-3 supplementation studies, a novel liquid chromatography–tandem mass spectrometry (LC–MS/MS) method was developed and validated for the simultaneous quantification of eicosapentaenoic acid (EPA), docosahexaenoic acid (DHA), eicosapentaenoyl-L-carnitine (EPAC), and docosahexaenoyl-L-carnitine (DHAC). Matrix effects were normalized using a background subtraction approach, and the analytes were extracted from blood plasma via simple protein precipitation with acetonitrile. Chromatographic separation was achieved within 5 min on a reverse-phase C18 column using a gradient mobile phase composed of ammonium acetate and acetonitrile. The limits of quantification of the method were 2 μM for EPA/DHA and 2 nM for EPAC/DHAC. The method exhibited high precision and accuracy, with coefficient of variation and bias values less than 10%. The stability tests confirmed that the analytes remained stable under various conditions, such as remaining up to 6 h at room temperature and refrigeration and undergoing three freeze–thaw cycles; however, the acylcarnitines were unstable during long-term storage. This method is simple, fast, and cost-effective; thus, it is suitable for high-throughput analysis of samples from clinical studies. A PUFA supplement study in healthy volunteers revealed a more pronounced increase in plasma EPAC and DHAC levels than in EPA and DHA levels; these results indicated that PUFA-derived acylcarnitines were potential novel and sensitive markers of PUFA intake.

**Graphical Abstract:**

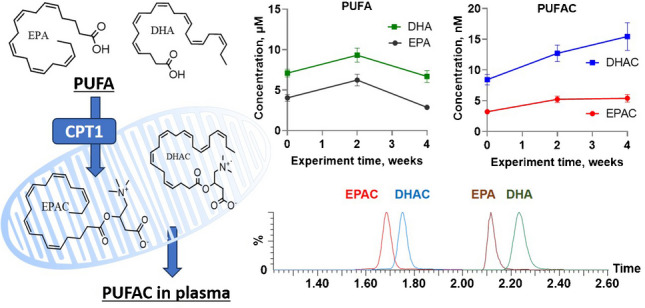

**Supplementary Information:**

The online version contains supplementary material available at 10.1007/s00216-025-05943-8.

## Introduction

The health benefits of omega-3 polyunsaturated fatty acids (PUFAs) are widely recognized in cardiometabolic disease management. In humans, dietary recommendations assume limiting the consumption amount of saturated fatty acids (FAs), which contributes to an increased risk of developing cardiovascular disease, type 2 diabetes, and inflammation [1]. A reduced risk of cardiovascular disease and type 2 diabetes, as well as other beneficial effects on health, are observed when saturated FAs in the diet are replaced with PUFAs [2, 3]. FAs and other lipid biomarkers are important epidemiological tools that help elucidate the relationship between the FA composition of the diet and health outcomes. Various lipid species measured in plasma and erythrocytes are used as biomarkers of saturated and unsaturated FA intake [4, 5]; however, changes in PUFA-containing phospholipid concentrations do not always correlate with dynamic changes in PUFA levels in the cardiac tissues. Since β-oxidation of long-chain FAs is an important source of ATP production in cardiac and skeletal muscle mitochondria, PUFAs are also metabolized by mitochondrial β-oxidation. The first step needed for entering mitochondria is the carnitine palmitoyltransferase-1 enzyme (CPT1), which catalyzes PUFA acylcarnitine (PUFAC) synthesis from carnitine and the corresponding acyl-CoA [6]. Some synthesized acylcarnitines are not metabolized but exit the mitochondria and appear in the blood. Therefore, the plasma concentrations of long-chain acylcarnitines are markers of mitochondrial fatty acid oxidation (FAO) status and reflect the cardiac content of acylcarnitines [7]. Moreover, recent findings indicate that long-chain acylcarnitines derived from omega-3 PUFAs, such as eicosapentaenoyl-L-carnitine (EPAC) and docosahexaenoyl-L-carnitine (DHAC), exert significantly lower cardiotoxic effects compared to saturated and monounsaturated acylcarnitine’s. These PUFA-derived acylcarnitines preserve cardiac function, maintain mitochondrial respiration, and do not impair insulin signaling or cell viability [8]. We hypothesize that PUFAC concentrations in plasma are valuable markers of the PUFA content in the heart. PUFAC could efficiently complement currently used markers of unsaturated FA lipid contents.

Several LC–MS/MS methods have been developed for measuring EPA, DHA, and various acylcarnitines in plasma, with substantial variability in sample preparation techniques (e.g., liquid-liquid extraction or protein precipitation), types of internal standards, matrix effect correction approaches, and analyte coverage [9–16]. A comparative overview of representative methods is provided in Table 1 (see Supplementary Materials). For example, while Wang et al. and Serafim et al. employed isotopically labeled internal standards with matrix effect correction via IS normalization, Zhou et al. used standard addition, and other methods relied on structural analogs or background subtraction. Chen and Zhang presented a method utilizing chemical isotope labeling with DnsHz to simultaneously analyze fatty acids and acylcarnitines in plasma samples [17]. Nevertheless, this method does not address the analysis of EPAC and DHAC, and the sample preparation for derivatizing fatty acids and acylcarnitines is time-consuming and less suitable for high-throughput workflows. To our knowledge, no validated analytical method has yet been published that enables the simultaneous, non-derivatized quantification of EPA, DHA, and their long-chain acylcarnitine derivatives (EPAC and DHAC). Given the potential of PUFA-derived acylcarnitines (PUFACs) as novel biomarkers for omega-3 fatty acid intake, the development of such a method would address a critical gap in lipidomics and nutritional monitoring.

In the absence of analyte-free biological matrices, accurate quantification of endogenous compounds such as EPA, DHA, and their acylcarnitines requires careful calibration strategies. In this study, we investigated the possibility of using a background subtraction method in routine LC–MS/MS analysis to compensate for the matrix effects of EPA, DHA, and their acylcarnitines. In background subtraction, the endogenous concentrations of the analytes in a pooled matrix are subtracted from the concentrations of the added standards [18]. This method enables matrix-matched calibration without requiring a blank matrix or labor-intensive standard addition. Notably, deuterated internal standards for EPAC and DHAC are not commercially available, limiting the applicability of IS-based matrix effect correction for these analytes. Therefore, background subtraction offered a practical and effective alternative for quantification.

## Materials and methods

### Reagents

Eicosapentaenoic acid (EPA; purity: 98%) was sourced from BLD Pharmatech (Reinbek, Germany), and docosahexaenoic acid (DHA; purity: 85%) was obtained from Biosynth (Bratislava, Slovakia). Eicosapentaenoic acid-d5 (EPA-d5; purity: 99%) and docosahexaenoic acid-d5 (DHA-d5; purity: 99%) were supplied by Cayman Chemical Corporation (Ann Arbor, MI, USA). Eicosapentaenoyl-L-carnitine (EPAC) and docosahexaenoyl-L-carnitine (DHAC) were synthesized in-house at the Latvian Institute of Organic Synthesis (Riga, Latvia). HPLC-grade acetonitrile, methanol, hydrochloric acid (HCl), sodium hydroxide (NaOH), and LiChropur ammonium acetate were acquired from Sigma–Aldrich (Schnelldorf, Germany). Formic acid (≥99%) was obtained from VWR Chemicals BDH (Leuven, Belgium). Pooled human plasma with EDTA was obtained from Innovative Research (Novi, MI, USA), and bovine serum albumin (fatty acid-free, used as a purified protein standard and served as a surrogate matrix) was supplied by Europa Bioproducts (Chelsworth, United Kingdom). Ultrapure water (resistance >18 MΩ) was generated using a Millipore Milli-Q purification system (Bedford, USA).

### Synthesis of polyunsaturated fatty acid acylcarnitines

**Scheme 1:**
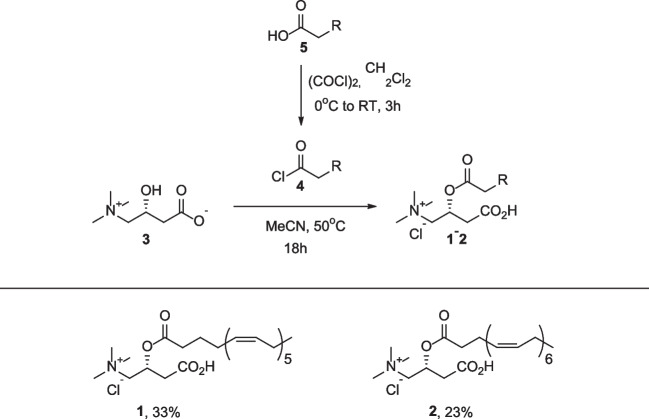
Synthesis of EPAC (**1**) and DHAC (**2**)

The synthesis of eicosapentaenoyl-L-carnitine (EPAC) and docosahexaenoyl-L-carnitine (DHAC) is shown in Scheme 1. EPAC (1) and DHAC (2) were synthesized through the acylation of carnitine (3) with acyl chlorides (4), derived from the corresponding unsaturated carboxylic acids (5) in the presence of oxalyl chloride. The target compounds 1 and 2 were obtained in 33% (263 mg) and 23% (177 mg) yields, respectively, using acetonitrile as the solvent. Yields were calculated based on the isolated mass relative to the theoretical yield corresponding to the molar amounts of starting materials (1.65 mmol for EPAC and 1.52 mmol for DHAC) [19]. A detailed description of the synthesis is provided in the supplementary material.

### Liquid chromatographic and mass spectrometric conditions

Analysis was performed on an ACQUITY UPLC system coupled with a Xevo TQ-S Micro triple-quadrupole mass spectrometer (Waters Corporation, Milford, MA, USA). Chromatographic separation of EPA, DHA, EPAC, DHAC, EPA-d5 (IS), and DHA-d5 (IS) was achieved using a BEH C18 column (2.1 mm × 50 mm, 1.7 μm) from Waters. The column temperature was maintained at 40 °C. The mobile phase consisted of 2 mM ammonium acetate adjusted to pH 4 with formic acid (A) and acetonitrile (B); these were used in gradient elution at a flow rate of 0.45 mL/min as follows: 0.0–0.5 min, 60% A; 0.7–2.5 min, 10% A; and 3.5–5.0 min, 60% B. The injection volume was 5 μL.

EPA, DHA, and their deuterated internal standards were detected via multiple reaction monitoring (MRM) in negative ESI ion mode, whereas acylcarnitines were detected in positive ESI mode, as shown in Table 1. The optimized mass spectrometric conditions were as follows: the capillary voltage was set to 1.5 kV; the ion source temperature was maintained at 140 °C; and the desolvation gas (N_2_, 1000 L/h) temperature was set to 600 °C. Argon was used as the collision gas, and nitrogen was employed as the cone gas at a flow rate of 50 L/h.

**Table 1:**
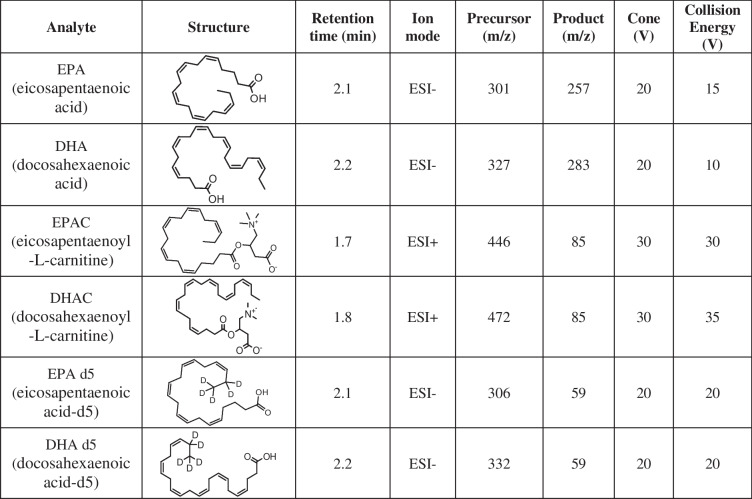
MRM and chromatographic conditions for the analytes and internal standards (IS)

### Data analysis

Waters MassLynx 4.1 and TargetLynx were used for data acquisition and processing. For EPA and DHA, calibration plots were constructed of the standard peak area ratio of the analyte/internal standard peaks, but for acylcarnitines, the peak areas were used. Weighted 1/x^2^ linear regression was used for all analytes. The concentrations of the QC samples were determined from the appropriate calibration line and used to calculate the bias and precision of the method with Microsoft Excel 365 (*Version 2403*). The quantitative results are expressed as the means ± standard errors of the means (SEMs), and graphical representations were created using Prism 3.0 software (GraphPad).

### Stock and working solutions

Stock solutions were individually prepared in methanol (MeOH) at final concentrations of 100 mM for EPA and DHA and 4 mM for acylcarnitines. The pooled stock solution was prepared by mixing the stock solutions in acetonitrile (ACN) at final concentrations of EPA, DHA 2000 μM, and acylcarnitines at 2 μM in ACN. Working solutions for calibration were prepared in 60% ACN at the following concentrations: 12, 24, 47, 92, 176, 323, and 557 μM for EPA and DHA and 12, 24, 47, 92, 176, 323, and 557 nM for acylcarnitines. Working solutions for quality control (QC) were prepared in 60% ACN at the following concentrations: 12, 36, 215, and 419 μM for EPA and DHA and 12, 36, 215, and 419 nM for acylcarnitines. Stable isotope-labeled internal standards, EPA-d5, and DHA-d5 were obtained at concentrations of 0.5 mg/mL in ethanol and diluted to a working concentration of 1 μM in acetonitrile (used as a precipitating solution). The stock solutions and working solutions were stored at −20 °C until use.

### Preparation of calibration samples using the background subtraction method

Each 50 μL plasma aliquot was individually spiked with 10 μL of standard working solutions to achieve final concentrations of 2, 4, 8, 15, 29, 54, and 93 μM for EPA and DHA and 2, 4, 8, 15, 29, 54, and 93 nM for acylcarnitines. The final concentrations for the quality control samples were 2 μM (LLOQ), 6 μM (LQC), 36 μM (MQC), and 70 μM (HQC) for fatty acids and the corresponding nanomolar concentrations for acylcarnitines.

Three parallel aliquots were prepared without the addition of a standard to subtract the endogenous background plasma concentration. To a 50 μL plasma sample, 10 μL of 60% ACN was added. The subtracted concentrations were subsequently used to construct the calibration lines.

### Study design and sample collection

This study was approved by the Riga Eastern Clinical University Hospital Support Fund Medical and Biomedical Research Ethics Committee (opinion No., 28/2022). A total of 13 volunteers agreed to participate in the study. They received 2 capsules of the food supplement Livol Extra 1000, which contains fish oil (380 mg of DHA and 880 mg of EPA). The plasma samples were collected before the study and 2 and 4 weeks after the daily intake of the supplement. Before the last blood sampling, 4 volunteers withdrew from the study for personal reasons. Analysis of EPA, DHA, and their acylcarnitines was carried out in 35 plasma samples from healthy volunteers. All samples were stored at −80 °C before analysis.

### Sample preparation for the analysis of free EPA, DHA, and acylcarnitines

The samples were prepared using a simple protein precipitation method. Specifically, 50 μL of plasma from healthy volunteers and 10 μL of 60% ACN were added to 300 μL of precipitation solution (acetonitrile containing 1 μM IS). Calibration standards and quality control samples (60 μL) were prepared by adding 300 μL of precipitation solution. The mixture was vortexed for 5 s and subsequently centrifuged at 10,000 rpm for 10 min. The resulting supernatant was collected and used for further analysis via LC–MS/MS.

### Sample preparation for the analysis of total EPA and DHA

Calibration standards for total triglyceride-derived EPA and DHA were prepared by serial dilution over the concentration range of 1.4–1000 µM using bovine serum albumin (BSA). Serum albumin or plasma samples (25 µL) were mixed with 150 µL of internal standard solution containing 5 µM d_5_-EPA and d_5_-DHA, or with acetonitrile (for blanks). Following Chester L. Bowen’s protocol [9], samples were vortexed, treated with 200 µL of 90:10 (v/v) acetonitrile/6 N HCl, and incubated at 104 °C for 45 min. After cooling, 200 µL of 90:10 (v/v) methanol/10 N NaOH was added, followed by a second 45-min incubation. After centrifugation, 200 µL of the supernatant was acidified with 100 µL of 6 N HCl and extracted with 600 µL of hexane. The hexane phase (200 µL) was evaporated and reconstituted in 300 µL of 50:50 (v/v) acetonitrile/water.

### Method validation

The method was validated according to the ICH bioanalytical method validation guidelines [20], and the parameters included selectivity, specificity, linearity, lower limit of quantitation (LLOQ), precision, accuracy, carry-over, recovery, matrix effect, and stability.

#### Specificity and selectivity

For endogenous compounds, the assessment of selectivity is complicated by the absence of an interference-free matrix. To evaluate the performance of the chromatography–tandem mass spectrometry method, we analyzed a blank sample prepared according to standard procedures (“Stock and working solutions” section, “Preparation of calibration samples using the background subtraction method” section, and “Sample preparation for the analysis of free EPA, DHA, and acylcarnitines” section) and substituted the plasma with the mobile phase (acetonitrile–2 mM ammonium acetate, pH 4 (90:10, v/v)).

Specificity in this study was ensured by employing a triple quadrupole mass spectrometer operating in multiple reaction monitoring (MRM) mode. To evaluate potential interferences, each substance was analyzed in the mobile phase at its LLOQ concentration (iLLOQ), and any interfering signals from other compounds were systematically assessed.

The selectivity and specificity of the internal standards (EPA-d5 and DHA-d5) were assessed across six different lots of human plasma to confirm the absence of endogenous interference with the mass transitions of the internal standards.

#### Lower limit of quantification

The instrumental LLOQ (iLLOQ) in the solvent (mobile phase) and the method LLOQ (mLLOQ) in the plasma were evaluated in accordance with the procedure described by Coglianese et al. [21]. For the determination of the instrumental LLOQ, samples were prepared as outlined in the “Sample preparation for the analysis of free EPA, DHA, and acylcarnitines” section, “Stock and working solutions” section and “Preparation of calibration samples using the background subtraction method” section, and acetonitrile–2 mM ammonium acetate (pH 4; 90:10, v/v) was substituted for plasma. To assess the accuracy and precision of the iLLOQ, calibration standards were prepared in the mobile phase at concentrations ranging from 0.5 to 23 μM for PUFAs and nanomolar concentrations for acylcarnitines. The iLLOQ was determined to be 0.5 μM for PUFA and 0.5 nM for PUFAC.

The method LLOQ (mLLOQ) was established using plasma samples prepared as previously described, with values of 2 μM for PUFAs and 2 nM for acylcarnitines. Pooled human plasma containing the lowest achievable level of interfering endogenous analytes was used for the calibration and quality control samples. In both cases, the LLOQ was defined as the lowest concentration that exceeded the endogenous level or baseline noise by at least threefold and could be quantified with acceptable accuracy and precision.

#### Linearity, precision, accuracy and carry-over

The calibration lines were fitted using linear regression with a 1/x^2^ weighting function and were considered acceptable if deviations were within 15% (or 20% for the LLOQ) for at least 75% of the calibration standards.

The within-run accuracy and precision were evaluated at the LLOQ, LQC, MQC, and HQC levels by analyzing 6 replicates. The between-run accuracy and precision were evaluated by analyzing six replicates of each QC concentration level over 3 days. Precision was defined as the variability among the replicate samples and was expressed as the coefficient of variation (CV, %). The CV was required not to exceed 15%, with the exception of the LLOQ level, where it was permitted to be ≤20%. The method accuracy was quantitatively expressed as relative bias. At each concentration, the level accuracy was required to be within ±15% of the nominal concentration, with the exception of the LLOQ, where it was permitted to be within ±20%.

Sample-to-sample carry-over was evaluated by injecting a blank sample immediately after three consecutive injections of the highest calibration standard. A blank sample was prepared according to standard procedures (“Stock and working solutions” section, “Preparation of calibration samples using the background subtraction method” section and “Sample preparation for the analysis of free EPA, DHA, and acylcarnitines” section), substituting plasma with the mobile phase (acetonitrile–2 mM ammonium acetate, pH 4 (90:10, v/v)), and proteins were precipitated using acetonitrile without internal standard. The analyte signal in the blank sample following the highest calibrator was required to be less than 20% of the analyte signal at the LLOQ level and less than 5% of the IS signal.

#### Stability

Stability was determined at the LQC (n = 3) and HQC (n = 3) levels under various conditions and evaluated using freshly prepared calibration standards. To ensure short-term stability, the plasma samples were stored on a benchtop at ambient temperature (+25 °C) for 6 and 24 h before processing. In addition, short-term stability was verified by storing plasma samples in a refrigerator (+4 °C) for 24 h. The stability of the analytes in the processed samples was evaluated at the autosampler temperature (+4 °C) after 23 h. The stability of the analytes in plasma after undergoing freeze–thaw cycles was evaluated after the QC samples were subjected to 3 freeze–thaw cycles from the freezer (−20 °C) to ambient temperature over a 7-day period. The long-term stability was tested by storing the plasma samples at −20 °C and −80 °C for 2 months. The stability of the working solutions was determined after 2 months of storage at −20 °C and compared with that of freshly prepared samples.

#### Matrix effect and recovery

To investigate the occurrence of matrix-induced variability, the matrix effect and recovery were evaluated at the LQC and HQC concentration levels by performing two replicates for the six lots of human plasma sources. The matrix effect was the ratio between the peak area of the analyte in the post-extraction samples and two standard samples, one prepared in an ACN solution and the other in bovine serum albumin (BSA). For comparable calculations, the endogenous level present in the plasma samples was subtracted. Extraction recovery was determined by comparing the peak area of the analytes from the pre-extracted plasma sample with that obtained from the post-extracted sample at the same concentration for the QC samples.

## Results

### Overcoming analytical challenges in LC–MS/MS quantification of endogenous EPA, DHA, and acylcarnitines in plasma

The objective of this study was to develop a rapid and high-throughput LC–MS/MS method for the quantitative determination of EPA, DHA, and acylcarnitine contents in human plasma without derivatization and complex sample preparation methods. The first challenge to overcome was the poor ionization of the fatty acids. The best results were achieved using C18 columns and ACN/ammonium acetate; this mobile phase significantly improved the ESI negative ionization. Second, the lack of a free-analyte matrix and deuterated internal standards causes difficulty in the preparation of a precise and accurate calibration curve. Method validation could not be performed in bovine serum albumin (BSA, purified, fatty acid-free) without appropriate internal standards due to matrix effects, and it can be expected that similarly purified human serum albumin as a surrogate matrix is not suitable. Furthermore, BSA proteins do not completely precipitate with ACN/MeOH, and upon contact with ammonium acetate present in the mobile phase, they form precipitates in the LC system, leading to increased pressure in the column. Finally, in the absence of a blank matrix, the lower quantification limit (LLOQ) is difficult to evaluate. The instrumental LLOQ (iLLOQ) in the solvent (mobile phase) and the method LLOQ (mLLOQ) in the plasma were evaluated according to Coglianese et al. [21]. The accuracy and precision were determined for both the iLLOQ and mLLOQ of the samples. During validation using the background subtraction method, matrices with the lowest possible levels of interfering endogenous analytes were selected. The amount of analyte added to the spiked samples was sufficient to achieve concentrations that were three times higher than the endogenous levels. The approach employed to resolve these problems is detailed in the following sections.

### Selectivity and specificity

**Fig. 1:**
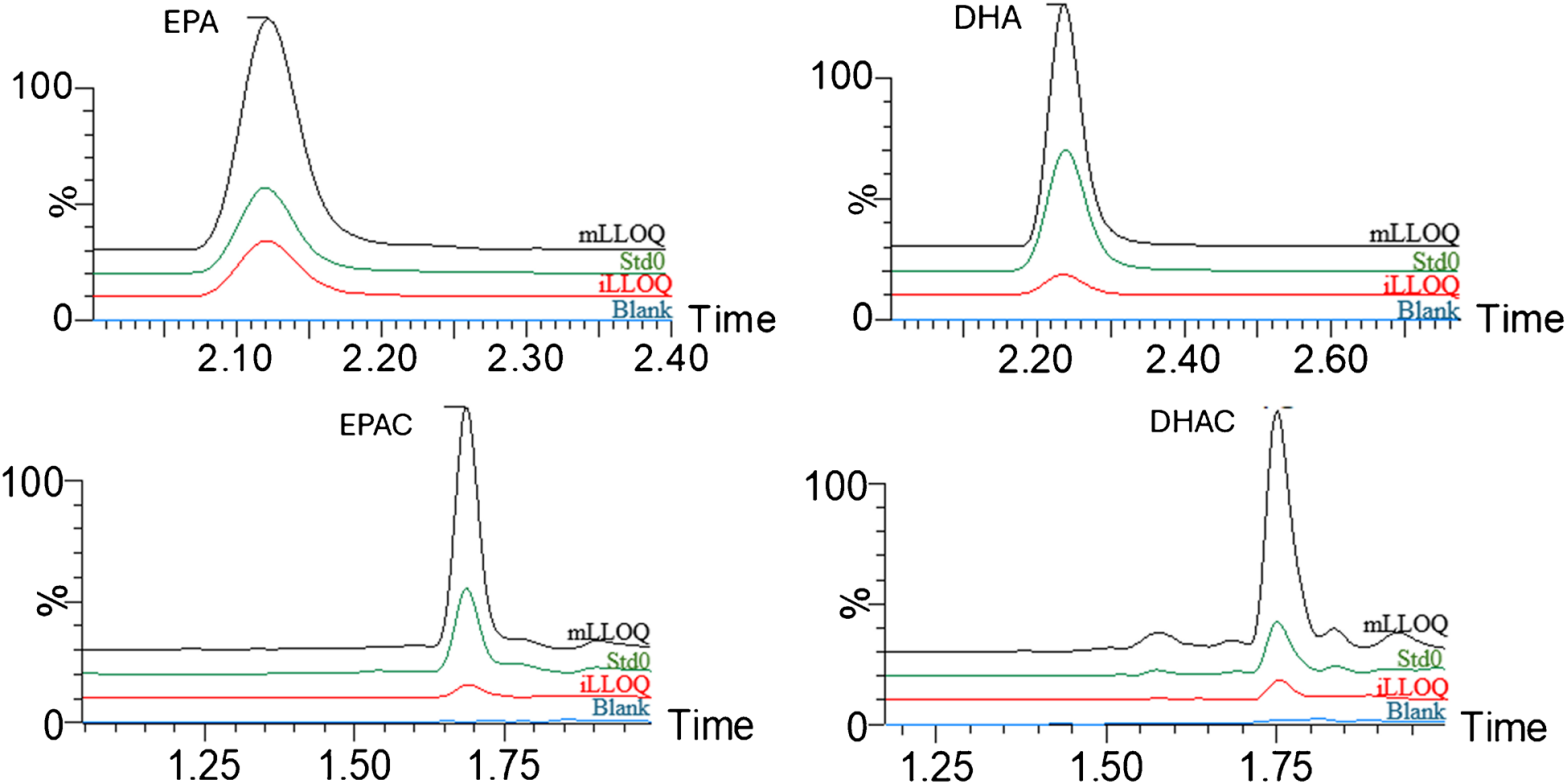
Representative MRM chromatograms of the blank sample in the mobile phase (Blank), instrumental LLOQ (iLLOQ), plasma sample containing analytes at the endogenous level (Std0) and method LLOQ (mLLOQ)

The selectivity experiment was conducted using blank samples prepared according to the protocol, with the solvent replacing the plasma. The MRM chromatograms in Fig. 1 show the blank sample in the mobile phase (blue), the lower limit of quantification in the mobile phase (iLLOQ; red), the lower limit of quantification in plasma (mLLOQ; black), and the endogenous signal of the plasma sample (Std0; green). These chromatograms confirmed that no potential interfering signals originated from the mobile phase or solvent at the retention times corresponding to the analytes.

Specificity was evaluated by assessing the presence of potential interfering components for each analyte. For this purpose, each analyte was individually prepared, and an iLLOQ sample was created in the mobile phase. No significant signals exceeding 20% of the analyte response at the iLLOQ were detected.

For all plasma blanks, the response at the retention time of the internal standards was <5% of their respective response. Additionally, during specificity testing, each internal standard was added individually to the sample, and no interfering signals were observed in any of the MRM channels.

### Lower limit of quantification

The quantification accuracy (Qbias %) and precision were evaluated based on the criteria outlined above. According to ICH validation guidelines [20], accuracy and precision are considered acceptable within 20%. The results obtained (see Table 2) demonstrate good accuracy and precision since the CV (%) and bias (%) at the LLOQ did not exceed 10%. For this approach to yield reproducible results, the increase in the background peak area after spiking with standards must exceed the method’s reproducibility limits, ideally by 15–20% of the background peak areas. Therefore, in this study, the LLOQ was defined as the minimum concentration that produced the smallest statistically significant increase over the background concentration (Std0), while maintaining acceptable accuracy and precision. Figure 1 illustrates this threshold (mLLOQ).

**Table 2:** Within- and between-run bias and precision for the QC samples in human plasma and iLLOQ in the mobile phase

PUFA	EPA					DHA				
	iLLOQ	mLLOQ	LQC	MQC	HQC	iLLOQ	mLLOQ	LQC	MQC	HQC
Concentration (μM)	0.5	2	5.9	35.8	69.8	0.5	2	5.9	35.8	69.8
Within-run bias (%)	3.1	−1.6	−5.3	−2.1	1.9	7.4	5.1	−2.5	−1	1.8
Within-run CV (%)	5.4	6.2	5.1	2.6	2.4	2.9	5.1	1.8	1.4	1.3
Between-run bias (%)	9.4	1.8	−3.9	−0.3	1.8	4.7	3.9	−2.4	0.1	2.2
Between-run CV (%)	6.5	9.6	4.2	2.6	2.3	3.6	5.2	2.3	1.8	1.3
PUFAC	EPAC					DHAC				
	iLLOQ	mLLOQ	LQC	MQC	HQC	iLLOQ	mLLOQ	LQC	MQC	HQC
Concentration (μM)	0.5	2	5.9	35.9	69.9	0.5	2	5.9	35.8	69.8
Within-run bias (%)	6.8	−5.6	−4.4	−1	2.1	−3.4	−6.2	−7.7	−1.8	1.7
Within-run CV (%)	5.1	1.9	1.6	1.6	1.5	8.2	6.3	2.7	2	1.6
Between-run bias (%)	−0.3	−2.6	−3.7	1.5	3.2	−0.3	−1.7	−5.2	0.9	2.8
Between-run CV (%)	7.9	3	2.2	2.4	1.5	9.9	6.2	3.2	2.8	1.7

### Linearity, precision, accuracy, and carry-over

The results are provided in Table 2. Similar to the LLOQ, the LQC, MQC, and HQC samples also demonstrated good accuracy (bias, %) and precision (CV, %). Across replicate samples (*n* = 6) at all concentration levels, the variation was less than 10% for both within- and between-run analyses.

The calibration lines were generated by spiking the authentic matrix with increasing concentrations of the analytes. The endogenous concentration was derived from the negative intercept with the x-axis. The resulting response lines were subsequently corrected for the endogenous (background) signal of the analytes in the authentic matrix, and the net differences were used to construct the calibration lines for the study samples. An example of the calibration line is provided in the Supplementary Information. The correlation coefficient (r^2^) for the between-run analyses was >0.998. Additionally, no carry-over between samples was observed following a single-blank injection.

### Stability

The data representing the stability of the analytes are listed in Table 3. These results indicate that the analytes remain stable in human plasma under bench-top conditions for up to 6 h and in a refrigerator (4 °C) for up to 24 h. Furthermore, they can undergo at least three freeze–thaw cycles and remain stable in acetonitrile-precipitated samples stored in an autosampler for at least 23 h. The stability was confirmed since the mean bias (%) from the nominal concentrations remained within the acceptance criterion of less than 15%. However, the analytes were not stable when stored for 2 months at both −20 °C and −80 °C. Similarly, the working solutions (60% in acetonitrile) were not stable when stored at −20 °C for 2 months.

**Table 3:** Bias (%) during stability assessment

	EPA		DHA		EPAC		DHAC	
	LQC	HQC	LQC	HQC	LQC	HQC	LQC	HQC
Bench-top (6 h)	−0.3	0.1	2.3	0.7	−10.5	−8.4	−13.1	−7
Bench-top (24 h)	0.2	0.9	1.8	−1.1	−18	−15	−16	−11
Freeze–thaw (3 cycles)	−9.8	−9	−12.1	−7.3	−4.3	4.8	−6.1	2.3
Autosampler (23 h)	2.8	1.3	2.6	0.1	−5.8	1.2	−10.3	0.3
Refrigerator +4 °C (24 h)	−7.8	0.5	−9.4	0.3	−2.8	5.1	−8.7	2
Long-term −20 °C (2 months)	12	16	−5	1.5	2	12	−36	−24
Long-term −80 °C (2 months)	5	13	−7	4	7	16	−28	−21
Working solutions −20 °C (2 months)	14	18	8	8	16	20	−24	−19

### Matrix effect and recovery

An IS-normalized matrix effect ranging from 98% to 113% was observed for fatty acids; these values were considered acceptable compared with spiked ACN and BSA. However, components extracted from the plasma matrix interfered with the ionization efficiency of acylcarnitine analytes. This interference resulted in matrix-induced ion enhancement and suppression, particularly when DHAC samples were prepared in BSA. These findings demonstrated that neither the BSA nor the ACN could serve as a suitable surrogate matrix for calibration curve construction.

The mean recovery of the analytes ranged from 81% to 100%, indicating satisfactory recovery performance. The detailed results regarding the matrix effects and recovery are provided in Table 4.

**Table 4:** Recovery and matrix effects

Compound	QC level	Matrix effect ± RSD, %		Recovery ± RSD, %
		ACN	BSA	
EPA	LQC	113 ± 2	111 ± 2	85 ± 5
	HQC	98 ± 3	100 ± 3	81 ± 6
DHA	LQC	107± 4	113 ± 4	94 ± 4
	HQC	101 ± 2	102 ± 2	87 ± 2
EPAC	LQC	459 ± 8	144 ± 8	100 ± 6
	HQC	445 ± 3	117 ± 3	94 ± 15
DHAC	LQC	181 ± 8	63 ± 8	94 ± 3
	HQC	233 ± 9	61 ± 9	88 ± 11

### EPA, DHA, EPAC, and DHAC levels in healthy volunteers

**Fig. 2:**
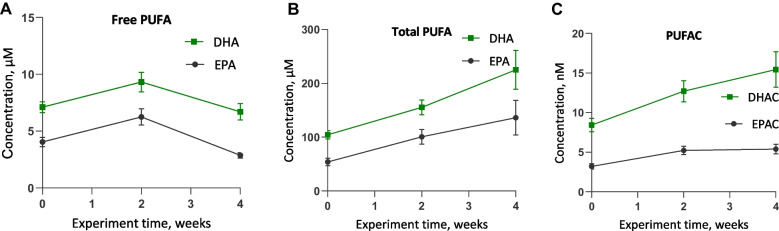
Plasma concentration–time profiles of free PUFA (**A**), total PUFA (**B**), and PUFAC (**C**) after 2 g/day of fish oil supplementation for 4 weeks in healthy volunteers. The plasma concentrations are expressed as the means from 9–13 volunteer plasma samples ± standard errors of the means (SEMs). Plasma samples were collected before (0) and after 2 and 4 weeks of supplement intake.

The validated method was applied to human plasma samples obtained from healthy volunteers (*n* = 13) following daily intake of 2 g of fish oil for 4 weeks. The plasma concentrations measured were within the analytical range of the proposed method. The time profile graphs are shown in Fig. 2, and detailed values are provided in the supplementary material.

Before fish oil administration, the plasma EPA concentrations in healthy volunteers were 4.0 ± 0.5 μM, the DHA concentration was 7.1 ± 0.5 μM, the EPAC concentration was 3.2 ± 0.3 nM, and the DHAC concentration was 8.4 ± 0.8 nM. The time profile graphs indicated that 2 weeks after fish oil intake, the plasma EPA and DHA concentrations increased by only 20% and 44%, respectively (Fig. 2A). However, after an additional 2 weeks of intake, both the EPA and DHA concentrations decreased below baseline at the start of the study. The DHAC and EPAC (Fig. 2C) concentrations increased by 60% after 2 weeks of supplement intake and remained at increased levels until the end of the study. These findings indicated that the increase in EPAC and DHAC plasma concentrations was more stable than that of EPA and DHA; therefore, PUFAC could serve as a more reliable biomarker of PUFA intake than the respective free fatty acids.

Interestingly, the total PUFA concentrations derived from triglycerides (Fig. 2B) showed a continuous and proportional increase throughout the supplementation period, like the pattern observed for DHAC and EPAC. This suggests that total PUFA concentrations may also serve as a more consistent biomarker of long-chain PUFA intake compared to the more variable free fatty acid levels.

To date, this is the first report of an LC–MS/MS method capable of simultaneously measuring the concentrations of EPA, DHA, and acylcarnitines in human plasma within a single run. Consequently, the developed method can be applied in population-scale studies to advance our understanding of the pharmacokinetics and pharmacodynamics of PUFAs.

## Conclusions

The validated LC–MS/MS method provided a rapid (5-min runtime), high-throughput solution for the simultaneous quantification of eicosapentaenoic acid (EPA), docosahexaenoic acid (DHA), and their acylcarnitines in human plasma. This method eliminated the need for derivatization or complex sample preparation, simplified the workflow, and maintained analytical precision.

The calibration lines showed good linearity (r^2^ > 0.998) across concentration ranges of 2–93 μM for EPA/DHA and 2–93 nM for EPAC/DHAC. The iLLOQ values were 0.5 μM for EPA/DHA and 0.5 nM for EPAC/DHAC, while the mLLOQ values were 2 μM for EPA/DHA and 2 nM for EPAC/DHAC. The precision (CV) and accuracy (bias) met ICH guidelines, with variation below 10% at all QC levels.

The method demonstrated satisfactory extraction recovery efficiencies (81% to 100%), but significant ion suppression and enhancement were observed in bovine serum albumin and acetonitrile matrices. Stability studies confirmed the analyte integrity under various conditions; the analytes could be stored up to 6 h at room temperature and under refrigerated conditions and could undergo three freeze–thaw cycles. However, the analytes were not stable for long-term storage in the freezer.

Plasma samples from healthy volunteers confirmed that the analyte concentrations in 95% of the clinical samples fell within the calibration range. This method is particularly well suited for clinical and pharmacokinetic studies of polyunsaturated fatty acids and derived acylcarnitines. According to our findings, in response to fish oil supplementation, the increase in plasma concentrations of PUFAC is more stable than that of free PUFAs. Therefore, PUFAC may serve as a more robust biomarker of PUFA intake than free unsaturated fatty acids. Further studies in larger cohorts are needed to confirm these initial findings.

To implement this method in another laboratory, revalidation is essential. Plasma samples with minimal endogenous analyte levels need to be selected to ensure accurate quantification. If necessary, blank matrices can be diluted, provided that recovery and matrix effect tests confirm that dilution does not introduce significant variability in analyte quantification. Even though the current method is optimized for fatty acids and their acylcarnitines, future adaptations could expand its applicability to other fatty acids, acylcarnitines, or biological matrices to further enhance its versatility.

## Supplementary Information

Below is the link to the electronic supplementary material.

Supplementary file1 (DOCX 124 KB)

## Data Availability

All data generated or analyzed during this study are included in the published article and its supplementary materials.

## Declarations

### Source of biological material

Blood samples were collected at Riga Eastern Clinical University Hospital.
