# Supplementary material for: LC–MS/MS-based simultaneous quantification of acylcarnitines, eicosapentaenoic acid, and docosahexaenoic acid: exploring potential biomarkers in human plasma
Source: Anal Bioanal Chem. 2025 Jun 7;417(19):4299–309. doi: 10.1007/s00216-025-05943-8 (PMC12283484; doi:10.1007/s00216-025-05943-8)
Supplement: Supplementary file 1 — Supplementary file1 (DOCX 124 KB) [file 216_2025_5943_MOESM1_ESM.docx]

**Supplementary Information**

**Table 1**

**Comparison of LC-MS/MS methods for PUFA and acylcarnitines analysis**

| Analytes | Matrix for calibration samples | Sample preparation technique | Internal Standards | Detects EPAC / DHAC | Sample Preparation Time | Approaches for matrix effect correction | Ref. |
| --- | --- | --- | --- | --- | --- | --- | --- |
| EPA, DHA | HSA | LLE (hexane) | [²H₅]-EPA, DHA | No | Medium | IS-normalized MF | Bowen CL 2010 |
| EPA, DHA, AA | Human plasma | LLE (chloroform) | d₈-Arachidonic acid | No | Medium | Matrix addition | Salm P, 2011 |
| EPA, DHA, ALA, DPA | Plasma | PP (ACN) | Chlorzoxazone | No | Short | Standard addition | Zhou B, 2019 |
| EPA, DHA | Human plasma | PP (ACN) | Nevirapine | No | Short | Background subtraction | Ghosh C, 2012 |
| EPA, DHA, ALA, ARA, LA | 80% MeOH | LLE (hexane/ isopropanol) | Fully deuterated IS | No | Medium | IS-normalized MF | Serafim V, 2019 |
| EPA, DHA | Serum | PP (ACN) | [²H₅]-EPA, DHA | No | Short | IS-normalized MF | Wang Y, 2023 |
| ARA, EPA, DHA | ACN | PP (ACN) | ARA-d₁₁ | No | Short | Not clearly stated; unclear if ARA-d₁₁ is used for normalization | Farczádi L, 2024 |
| AcCarn | MeOH | PP (0.3% FA in ACN) | Fully deuterated IS | No | Short | IS-normalized | Luna C, 2022 |
| FAs, AcCarn | Plasma | PP (ACN) Derivatization (DnsHz) | Heavy labeled DnsHz analogs | No | Long | One-on-one correction of endogenous  analytes. | Chen G yuan, 2020 |
| AcCarn | Stripped human serum | PP ACN | Deuterated internal standard, structurally similar analogs | No | Short | Effect largely corrected by internal standards | Meikopoulos T, 2022 |
| EPA, DHA, EPAC, DHAC | Plasma | PP (ACN) | Deuterated IS for EPA, DHA, EPAC, DHAC | Yes | Short | Background subtraction | *This study |

EPA – eicosapentaenoic acid; DHA – docosahexaenoic acid; EPAC – eicosapentaenoyl-L-carnitine; DHAC – docosahexaenoyl-L-carnitine; AA/ARA – arachidonic acid; ALA – alpha-lipoic acid; DPA – docosapentaenoic acid; LA – linoleic acid; AcCarn – acylcarnitines; FAs – fatty acids; LLE – liquid-liquid extraction; PP – protein precipitation; HSA – human serum albumin; *method developed in this study.

*Synthesis of polyunsaturated fatty acid acylcarnitines*

**(*R*)-3-Carboxy-2-(((5*Z*,8*Z*,11*Z*,14*Z*,17*Z*)-icosa-5,8,11,14,17-pentaenoyl)oxy)-*N,N,N*-trimethylpropan-1-aminium chloride** **(1)**

The solution of eicosapentaenoic acid (500 mg, 1.65 mmol) in anhydrous CH_2_Cl_2_ (5 mL) was cooled to 0 ˚C and oxalyl chloride (0.43 mL, 4.96 mmol) was added dropwise. The reaction mixture was stirred at 0 ˚C for 10 min. Then at room temperature for 3h. Volatiles were evaporated. Obtained eicosapentaenoyl chloride (530 mg, 1.65 mmol) was dissolved in anhydrous MeCN (5 mL), and *l*-carnitine (266 mg, 1.65 mmol) was added. The mixture was stirred at 50 ˚C for 18 h. Volatiles were evaporated. The crude was purified by reversed-phase column chromatography on C-18-SH modified silica gel (eluent 0.1% HCl/H_2_O-MeCN grad.) to yield a yellow amorphous solid (263 mg, 33 %). [α]_D_^20^ = –5.73 (*c*=1.5, CHCl_3_). IR (neat; cm^-1^): 3025 (O-H), 1732 (C=O). ^1^H NMR (400 MHz, MeOD) δ 5.66 – 5.59 (m, 1H), 5.45 – 5.26 (m, 10H), 3.87 (dd, *J* = 14.4, 8.5 Hz, 1H), 3.71 (d, *J* = 14.4 Hz, 1H), 3.20 (s, 9H), 2.91 – 2.69 (m, 10H), 2.44 – 2.38 (m, 2H), 2.19 – 2.04 (m, 4H), 1.75 – 1.66 (m, 2H), 0.97 (t, *J* = 7.6 Hz, 3H). ^13^C NMR (100 MHz, MeOD) δ 173.9, 172.4, 132.8, 130.1, 129.8, 129.5, 129.23, 129.17, 129.16, 129.08, 128.91, 128.2, 69.4, 66.3, 54.5, 37.7, 34.5, 27.5, 26.6, 26.5, 26.4, 25.6, 21.5, 14.7. HRMS-ESI (m/z) calculated for C_27_H_44_NO_4_ [M]^+^ 446.3270, found: 446.3275.

**(*R*)-3-Carboxy-2-(((4*Z*,7*Z*,10*Z*,13*Z*,16*Z*,19*Z*)-docosa-4,7,10,13,16,19-hexaenoyl)oxy)-*N,N,N*-trimethylpropan-1-aminium chloride (2)**

The solution of cis-4,7,10,13,16,19-docosahexaenoic acid (500 mg, 1.52 mmol) in anhydrous CH_2_Cl_2_ (5 mL) was cooled to 0 ˚C and oxalyl chloride (0.40 mL, 4.57 mmol) was added dropwise. The reaction mixture was stirred at 0 ˚C for 10 min. Then at room temperature for 3h. Volatiles were evaporated. Obtained cis-4,7,10,13,16,19-docosahexaenoyl chloride chloride (528 mg, 1.52 mmol) was dissolved in anhydrous MeCN (5 mL) and *l*-carnitine (245 mg, 1.52 mmol) was added. The mixture was stirred at 50 ˚C for 18 h. Volatiles were evaporated. The crude was purified by reversed-phase column chromatography on C-18-SH modified silica gel (eluent 0.1% HCl/H_2_O-MeCN grad.) to yield an air-sensitive yellow oil (177 mg, 23 %). [α]_D_^20^ = –5.48 (*c*=1.0, CHCl_3_). IR (neat; cm^-1^): 3013 (O-H), 1739 (C=O). ^1^H NMR (400 MHz, MeOD) δ 5.66–5.59 (m, 1H), 5.46–5.26 (m, 12H), 3.91–3.81 (m, 1H), 3.74–3.67 (m, 1H), 3.20 (s, 9H), 2.92–2.72 (m, 12 H), 2.50–2.38 (m, 4H), 2.13–2.04 (m, 2H), 0.97 (t, *J*=7.6 Hz, 3H). ^13^C NMR (100 MHz, MeOD) δ 173.6, 172.4, 132.8, 130.7, 129.5, 129.3, 129.2, 129.1, 129.0, 128.9, 128.8, 128.2, 69.4, 66.3, 54.6, 37.7, 35.0, 26.58, 26.55, 26.4, 23.5, 21.5, 14.7. HRMS-ESI (m/z) calculated for C_29_H_46_NO_4_ [M]^+^ 472.3427, found: 472.3417.


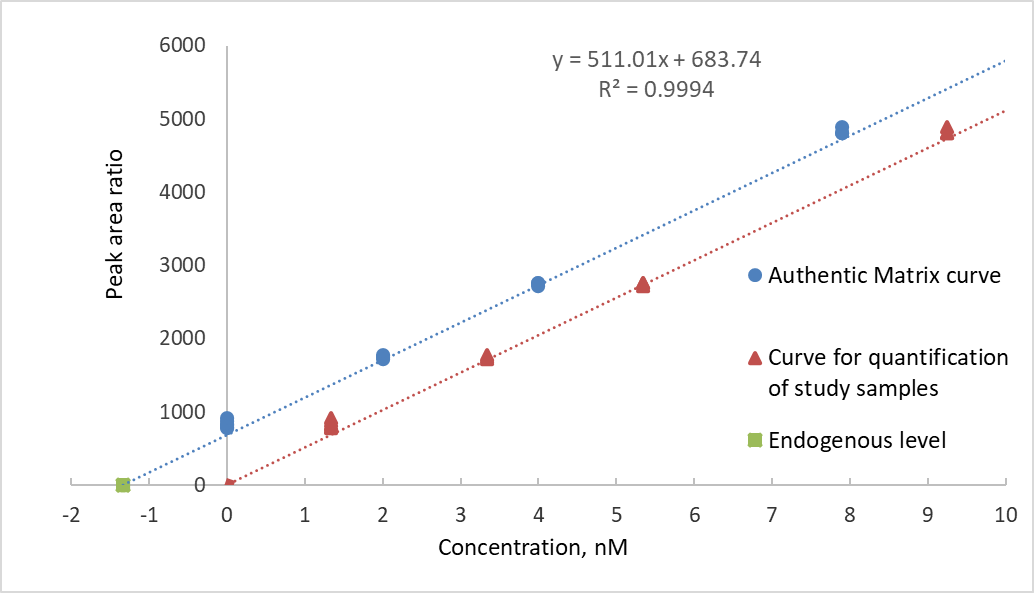


**Fig. 1.** Example calibration line for EPAC using the background subtraction method to account for endogenous levels.

**Table 2**

Concentration profiles of **free EPA**, **free DHA**, **EPAC**, and **DHAC** in the plasma of healthy volunteers (n=13) were analyzed during fish oil administration (2 g/day) over a treatment period of 0–4 weeks.

| Sample # | **EPA, μg/mL** | | | **DHA, μg/mL** | | | **EPA-AC, ng/mL** | | | **DHA-AC, ng/mL** | | |
| --- | --- | --- | --- | --- | --- | --- | --- | --- | --- | --- | --- | --- |
|  | **0 weeks** | **2 weeks** | **4 weeks** | **0 weeks** | **2 weeks** | **4 weeks** | **0 weeks** | **2 weeks** | **4 weeks** | **0 weeks** | **2 weeks** | **4 weeks** |
| 1 | 1.3 | 2.7 | 2.1 | 4.8 | 6.3 | 5.7 | 1.1 | 2.0 | 2.2 | 5.2 | 5.0 | 6.6 |
| 2 | 4.2 | 6.6 | 2.1 | 8.4 | 10.6 | 5.5 | 3.3 | 4.7 | 4.3 | 8.8 | 12.4 | 13.9 |
| 3 | 7.1 | 7.2 | 2.9 | 7.8 | 9.2 | 6.5 | 5.6 | 6.8 | 6.7 | 8.6 | 15.9 | 17.1 |
| 4 | 3.1 | 2.5 | 2.4 | 4.3 | 4.9 | 4.8 | 2.1 | 2.1 | 3.6 | 4.9 | 5.5 | 9.2 |
| 5 | 1.8 | 12.4 | - | 5.6 | 15.8 | - | 2.2 | 7.2 | - | 5.4 | 16.3 | - |
| 6 | 5.8 | 5.8 | 3.9 | 9.8 | 11.6 | 10.7 | 2.7 | 5.0 | 5.1 | 8.7 | 19.7 | 15.6 |
| 7 | 4.1 | 4.6 | - | 7.2 | 7.4 | - | 2.8 | 5.7 | - | 6.3 | 14.3 | - |
| 8 | 4.3 | 6.0 | 2.6 | 7.1 | 5.0 | 4.8 | 3.0 | 6.2 | 7.2 | 7.7 | 9.5 | 17.6 |
| 9 | 5.1 | 7.4 | 3.4 | 9.2 | 11.7 | 5.9 | 3.9 | 5.7 | 7.4 | 12.1 | 18.0 | 22.5 |
| 10 | 3.4 | 7.7 | 2.4 | 5.5 | 12.6 | 6.2 | 2.4 | 5.0 | 4.7 | 6.4 | 8.6 | 8.9 |
| 11 | 4.1 | 5.0 | - | 9.0 | 8.2 | - | 4.0 | 4.5 | - | 11.8 | 11.0 | - |
| 12 | 3.9 | 4.8 | 4.0 | 7.4 | 8.9 | 10.2 | 4.5 | 4.2 | 7.3 | 15.4 | 11.0 | 27.5 |
| 13 | 4.3 | 8.4 | - | 6.2 | 8.9 | - | 4.1 | 8.9 | - | 8.2 | 18.0 | - |
| **avg** | **4.0** | **6.2** | **2.9** | **7.1** | **9.3** | **6.7** | **3.2** | **5.2** | **5.4** | **8.4** | **12.7** | **15.4** |
| SD | 1.5 | 2.6 | 0.7 | 1.7 | 3.1 | 2.2 | 1.2 | 1.9 | 1.9 | 3.1 | 4.8 | 6.8 |
| **SEM** | **0.4** | **0.7** | **0.2** | **0.5** | **0.9** | **0.7** | **0.3** | **0.5** | **0.6** | **0.9** | **1.3** | **2.3** |

**Table 3**

The plasma concentration profiles of **EPA** and **DHA** in their **total triglyceride-bound** forms were analyzed in healthy volunteers (n = 13) during fish oil supplementation (2 g/day) over a 0–4 weeks treatment period.

| Sample # | **EPA, μg/mL** | | | **DHA, μg/mL** | | |
| --- | --- | --- | --- | --- | --- | --- |
|  | **0 weeks** | **2 weeks** | **4 weeks** | **0 weeks** | **2 weeks** | **4 weeks** |
| 1 | 11 | 31 | 43 | 61 | 69 | 99 |
| 2 | 48 | 96 | 89 | 113 | 169 | 194 |
| 3 | 92 | 109 | 103 | 116 | 124 | 182 |
| 4 | 38 | 39 | 68 | 70 | 101 | 161 |
| 5 | 31 | 159 | - | 107 | 180 | - |
| 6 | 91 | 101 | 148 | 140 | 203 | 292 |
| 7 | 74 | 86 | - | 132 | 162 | - |
| 8 | 41 | 105 | 122 | 64 | 108 | 147 |
| 9 | - | 76 | 208 | - | 133 | 299 |
| 10 | 49 | 109 | 87 | 112 | 198 | 193 |
| 11 | 34 | 61 | - | 91 | 121 | - |
| 12 | 71 | 119 | 358 | 135 | 232 | 460 |
| 13 | 65 | 216 | - | 110 | 219 | - |
| **avg** | **54** | **100** | **136** | **104** | **155** | **225** |
| SD | 25 | 49 | 96 | 27 | 50 | 109 |
| **SEM** | **7** | **13** | **32** | **8** | **14** | **36** |
